# A Phase II Study of Pelareorep (REOLYSIN^®^) in Combination with Gemcitabine for Patients with Advanced Pancreatic Adenocarcinoma

**DOI:** 10.3390/cancers10060160

**Published:** 2018-05-25

**Authors:** Devalingam Mahalingam, Sanjay Goel, Santiago Aparo, Sukeshi Patel Arora, Nicole Noronha, Hue Tran, Romit Chakrabarty, Giovanni Selvaggi, Andres Gutierrez, Matthew Coffey, Steffan T. Nawrocki, Gerard Nuovo, Monica M. Mita

**Affiliations:** 1 Division of Hematology/Oncology, Robert H. Lurie Comprehensive Cancer Center, Northwestern University, Chicago, IL 60611, USA; 2 Cancer Therapy and Research Center, University of Texas Health Science Center, San Antonio, TX 78229, USA; aroras@uthscsa.edu; 3 Montefiore Medical Center, New York, NY 10467, USA; sgoel@montefiore.org (S.G.); santiaparo@hotmail.com (S.A.); 4 Oncolytics Biotech Inc., Calgary, AB T2N 1X7, Canada; NNoronha@oncolytics.ca (N.N.); htran@oncolytics.ca (H.T.); rchakrabarty@oncolytics.ca (R.C.); giovanni.selvaggi@BMS.com (G.S.); AGutierrez@oncolytics.ca (A.G.); MCoffey@oncolytics.ca (M.C.); 5 Department of Medicine, Division of Translational and Regenerative Medicine, University of Arizona Cancer Center, Tucson, AZ 85724, USA; snawrocki@email.arizona.edu; 6 Comprehensive Cancer Center, Ohio State University, Columbus, OH and Phylogeny, Inc., Powell, OH 43065, USA; jerrynuovo@yahoo.com; 7 Samuel Oschin Comprehensive Cancer Institute, Los Angeles, CA 90048, USA; monica.mita@cshs.org

**Keywords:** REOLYSIN^®^, pelareorep, reovirus, immuno-oncolytic virus, pancreatic cancer, PD-L1

## Abstract

Pancreatic ductal adenocarcinoma (PDAC) has a poor prognosis, with 1 and 5-year survival rates of ~18% and 7% respectively. FOLFIRINOX or gemcitabine in combination with nab-paclitaxel are standard treatment options for metastatic disease. However, both regimens are more toxic than gemcitabine alone. Pelareorep (REOLYSIN^®^), a proprietary isolate of reovirus Type 3 Dearing, has shown antitumor activity in clinical and preclinical models. In addition to direct cytotoxic effects, pelareorep can trigger antitumor immune responses. Due to the high frequency of RAS mutations in PDAC, we hypothesized that pelareorep would promote selective reovirus replication in pancreatic tumors and enhance the anticancer activity of gemcitabine. Chemotherapy-naïve patients with advanced PDAC were eligible for the study. The primary objective was Clinical Benefit Rate (complete response (CR) + partial response (PR) + stable disease (SD) ≥ 12 weeks) and secondary objectives include overall survival (OS), toxicity, and pharmacodynamics (PD) analysis. The study enrolled 34 patients; results included one partial response, 23 stable disease, and 5 progressive disease. The median OS was 10.2 months, with a 1- and 2-year survival rate of 45% and 24%, respectively. The treatment was well tolerated with manageable nonhematological toxicities. PD analysis revealed reovirus replication within pancreatic tumor and associated apoptosis. Upregulation of immune checkpoint marker PD-L1 suggests future consideration of combining oncolytic virus therapy with anti-PD-L1 inhibitors. We conclude that pelareorep complements single agent gemcitabine in PDAC.

## 1. Introduction

Pancreatic cancer remains one of the most lethal cancers, ranking as the fourth leading cause of cancer death in both men and women [1]. Pancreatic cancer continues to be characterized by late stage of presentation, lack of effective chemotherapy, and devastating outcomes, with a 5-year survival rate of 7% for all stages of the disease [1].

In the last two decades, gemcitabine has been the mainstay of first-line therapy for unresectable locally advanced or metastatic pancreatic cancer [2]. Recently, FOLFIRINOX (Folinic Acid, 5-FU, Irinotecan, and Oxaliplatin) or gemcitabine in combination with nab-paclitaxel have shown greater efficacy than gemcitabine alone and are now standard treatment options for metastatic disease [3,4,5]. FOLFIRONOX improved median overall survival (OS) to 11.1 months with 1-year survival rate of 48.4% when compared to gemcitabine alone with 6.8 months and 20.6%, respectively [3]. The addition of nab-paclitaxel to gemcitabine improved median OS to 8.5 months with a 1-year survival rate of 35% when compared to gemcitabine alone with 6.7 months and 22%, respectively [4]. Despite the improved efficacy of these new regimens, both regimens are more toxic than gemcitabine alone, often limiting their use to patients with good performance status. As a result, gemcitabine monotherapy remains the standard treatment for patients who are not candidates for more intensive chemotherapy regimens, and therefore, better tolerated treatment regimens are needed for patients with advanced pancreatic cancer [6]. New treatment paradigms are desperately needed, including novel therapeutic targets aimed to inhibit the key oncogenic signals in pancreatic cancer, in particular the presence of constitutive activation of RAS in approximately 70–90% of pancreatic cancers [7,8,9]. 

Pelareorep (REOLYSIN^®^, Oncolytics Biotech Inc., Calgary, AB, Canada) is a proprietary isolate of reovirus Type 3 Dearing. This unmodified oncolytic reovirus has been extensively evaluated in preclinical models and clinical studies, and preferentially targets cancer cells based on their higher rates of cell division, which differs from that of normal cells (as reviewed in [10]). The dual mechanism of action of reovirus involves the selective lysis of tumor cells and induction of an antitumor immunity. The selective permissiveness of cancer cells to reovirus replication and lysis is dependent on a number of factors, including: (1) defective double-stranded RNA activated protein kinase (PKR) signaling; (2) RAS activation and/or mutations in upstream and downstream RAS-effector proteins that downregulate the interferon (IFN)-induced antiviral response; (3) mutations in key tumor suppressor genes and oncogenes (e.g., p53 and ataxia telangiectasia mutated (ATM)); and (4) cellular stress resulting from chemotherapy and radiation therapy [10,11,12,13,14,15]. Tumor cells infected with pelareorep release inflammatory cytokines, thereby activating natural killer (NK) cells, dendritic cells, and T cells, which aid in immune-mediated cancer cell death [16]. Following pelareorep enabled tumor cell lysis, viral- and tumor-associated antigens are released and taken up by antigen presenting cells, which then educate the immune system to recognize and kill cancer cells. A robust adaptive antitumor immune response follows, which allows for the elimination of tumor cells, constant cancer cell surveillance, and increased survival [17,18,19,20,21,22,23].

Reovirus has been shown to preferentially infect, induce endoplasmic reticulum (ER) stress, and kill RAS-activated pancreatic cancer cells [24,25]. Preclinical and clinical data suggest that the use of immune-modulating chemotherapeutic drugs in combination with reovirus may enhance the anticancer effects of reovirus by attenuating the antibody response and allowing enhanced viral replication and circulation for extended periods of time [26]. In a phase I study, pelareorep at the dose of 1 × 10^10^ TCID_50_ intravenously (IV) was safely combined with gemcitabine, with the most common side effects classified as grade 1 or 2, involving mild “flu-like” symptoms and manageable gastrointestinal symptoms [27,28].

Due to the high frequency of RAS mutations in pancreatic adenocarcinoma (PDAC), we hypothesized that pelareorep would enhance the anticancer activity of gemcitabine, and through immune modulation, it would ultimately augment the therapeutic efficacy of the combination. We therefore performed a phase II study to evaluate the efficacy of this regimen in patients with advanced pancreatic cancer. 

## 2. Results

### 2.1. Patient Characteristics

A total of 34 patients were enrolled from May 2010 to September 2012. The median age was 66 years (range 48–85) with 53% of patients more than or equal to 65 years of age. Fifty-three percent were male. Seventy-one percent were Caucasian, 12% Black, 3% Hispanic, 3% Asian, and 94% had an Eastern Cooperative Oncology Group (ECOG) of 0–1. Ninety-one percent of patients had metastatic disease; 65% with liver metastases, 18% with peritoneal involvement, and 6% lung metastases (Table 1.)

### 2.2. Efficacy

**Figure 1:**
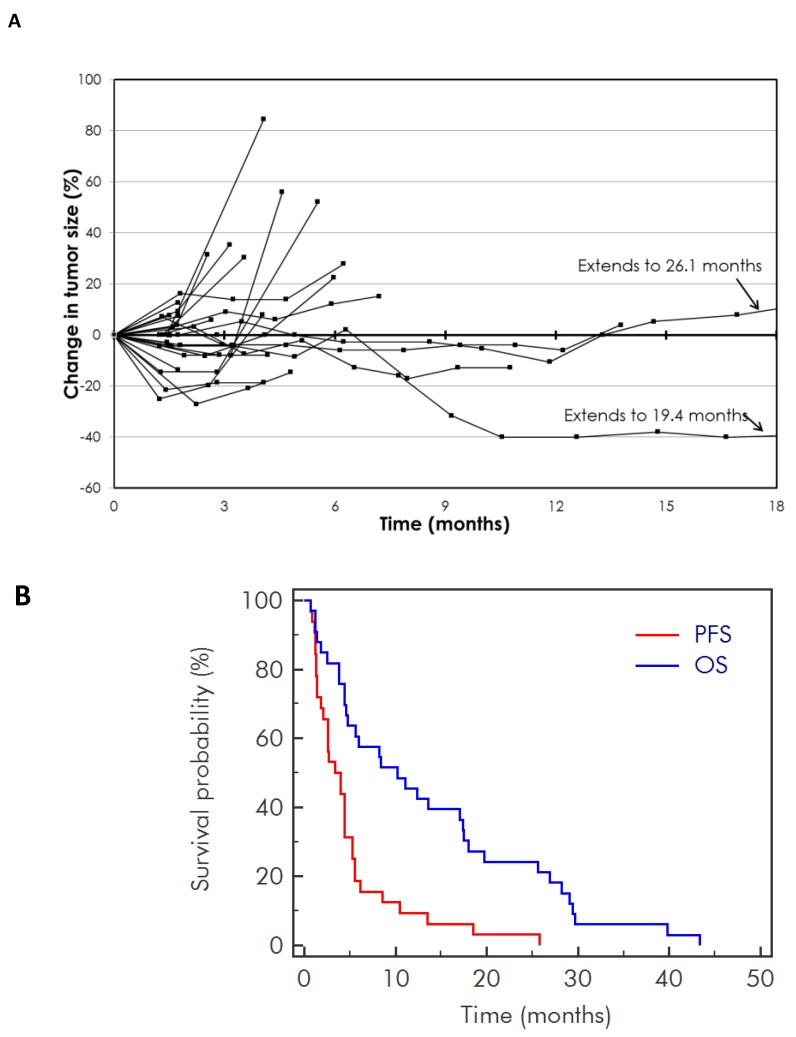
Survival of patients with advanced pancreatic cancer receiving pelareorep in combination with gemcitabine. (**A**) Spider plot showing the change in tumor size at each 6 week time point for 29 patients; (**B**) progression free survival and overall survival for all patients on study.

The median number of cycles received was four, with 29 patients evaluable for response. There was one confirmed partial response (PR) at 42 weeks. Twenty-three patients had stable disease (SD) and five patients had progressive disease (PD) as the best response. Overall, 17 patients had a clinical benefit measured as PR/SD at 12 weeks or more with a clinical benefit rate (CBR) of 58% [one PR (3%) and 16 SD (55%)]. For all evaluable patients on study, the percentage change in response and duration of response is outlined in the spider plot (Figure 1A). Seventy percent of patients had a CA19.9 decrease greater than 20% from baseline. The median progression free survival (PFS) was 3.4 months (95% CI from 2.1 to 4.4 months). The median OS was 10.2 months (95% CI from 4.8 to 17.4 months), with a 1-year and 2-year survival of 45% and 25%, respectively (Figure 1B). Median duration of follow up was 2 years. Fifty-three percent of patients received chemotherapy after progression, including 12% nab-paclitaxel. Median OS for patients with liver metastases was 4.8 months (*n* = 20, 95% CI from 3.8 to 10.2 months), and without liver metastases was 18.0 months (*n* = 13, CI from 12.4 to 28.2 months) with *p* = 0.05 (Supplemental Figure S1).

### 2.3. Toxicity

Overall, the treatment was well tolerated with manageable toxicities (Table 2). The most frequent nonhematological toxicities of all grades included fatigue (71%), fever (56%), flu-like symptoms or chills (51%), dyspnea (50%), edema (33%), anorexia/weight loss (33%), nausea (29%), vomiting (24%), and diarrhea (24%). In the majority of cases they were self-limited and short-lived or treatable with symptomatic therapy. Grade 3 nonhematologic toxicities were limited to fatigue (9%), dyspnea (6%), and elevated aspartate aminotransferase (AST) (6%). Hematological toxicities of all grades included anemia (35%), neutropenia (32%), and thrombocytopenia (15%) with grade 3–4 toxicities including anemia (27%), neutropenia (27%), and thrombocytopenia (6%). Two patients had febrile neutropenia (6%). 

### 2.4. Pharmacodynamic Analysis

**Figure 2:**
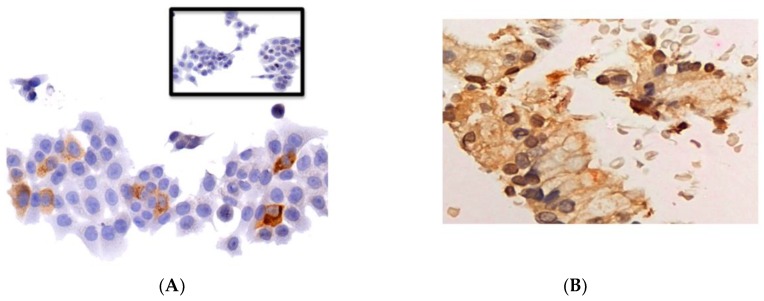

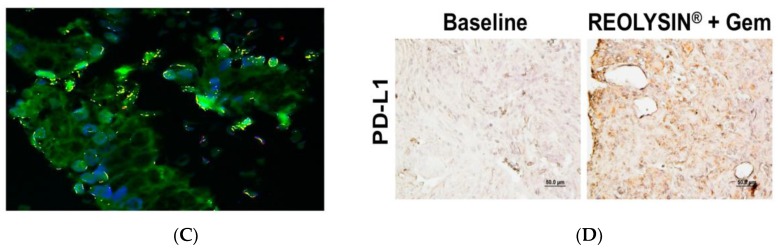
Tumor biopsy from the primary pancreatic tumor of a single patient with a KRAS G12D mutation following treatment with pelareorep and gemcitabine. (**A**) Immunohistochemistry (IHC) analysis of HCT116 colon cancer cells treated with pelareorep serves as a positive control for reoviral protein replication (brown). The uninfectecd HCT116 cell line is the negative control (inset); (**B**) IHC shows positive staining for reoviral protein and activated caspase-3 protein localized to cancer cells; (**C**) Fluorescent in situ hybridization (FISH) demonstrates coexpression of reoviral protein and caspase-3 consistent with productive lytic infection in the patient’s tumor. Yellow indicates colocalizaiton of reovirus (green) and caspase-3 (red) in the same cancer cells; (**D**) analysis of the tumor also reveals upregulation of programmed death ligand 1 (PD-L1) on IHC following pelareorep therapy (left panel represents the baseline; right panel represents pelareorep with gemcitabine). Scale bar: 50 µm.

KRAS mutation analysis, via Foundation Medicine genomic profile testing, was obtained from 15 of 34 patients on study. Of them, 12 (80%) had KRAS mutation (seven patients with G12D, four patients with G12V, and one patient with G12C KRAS mutations). A list of patient’s genetic aberration, location of archival tumor, and survival of the patient is provided in Supplementary Table S1. A single patient who had a KRAS G12D mutation received an on-treatment biopsy (Figure 2). This patient received a total of 27 cycles of treatment, and achieved SD as the best response and an OS of 29.1 months. Immunohistochemistry (IHC) stains showed positivity for reoviral protein and activated caspase-3 protein localized to the cancer cells (Figure 2B). Fluorescent in situ hybridization (FISH) demonstrated coexpression of reoviral protein and caspase-3, consistent with productive lytic infection in the patient’s tumor (Figure 2C). Analysis of the tumor also revealed upregulation of programmed death ligand 1 (PD-L1) on IHC following pelareorep therapy (Figure 2D).

## 3. Discussion

In this study, the combination of pelareorep plus gemcitabine was well tolerated, with most common toxicities being mild fatigue, fever, and flu-like symptoms. Given the favorable toxicity profile, this regimen may be complementary to gemcitabine monotherapy, especially in pancreatic cancer patients who are often not candidates for intensive chemotherapy regimens. Pelareorep in combination with gemcitabine resulted in a CBR of 58% at 12 weeks, including one prolonged PR of 42 weeks. The 3.4 month median PFS, 10.2 month median OS, and 1- and 2-year survival of 45% and 25%, respectively, is on par with a phase II single arm, nonrandomized study of gemcitabine (1000 mg/m^2^) and nab-paclitaxel (100, 125, or 150 mg/m^2^), which demonstrated an overall median PFS and OS of 7.1 months and 10.3 months, respectively [29]. Survival data from both of these studies are amongst the highest observed for phase II clinical trials in patients with advanced pancreatic adenocarcinoma. However, the gemcitabine and nab-paclitaxel regimen had increased incidence of treatment-related adverse events of any grade compared to the pelareorep study, with 98% experiencing anemia, 91% leukopenia, 89% neutropenia, 83% thrombocytopenia, 76% fatigue, 76% alopecia, and 63% sensory neuropathy. Also, dose-limiting toxicities (sepsis and neutropenia) were identified in the gemcitabine and nab-paclitaxel study [29].

The pelareorep regimen was also favorable in comparison with historical survival data from two large randomized phase III studies in metastatic pancreatic cancer comparing single agent gemcitabine (at the time standard of care treatment) versus gemcitabine/nab-paclitaxel in the MPACT study [4] or FOLFIRINOX in the ACCORD 11 study [3], where the gemcitabine arm in both studies showed a median OS and 1-year and 2-year survival of approximately 6.8 months, 20–22% and 2–5%, respectively [30]. Although in the pelareorep study, where the majority of the patients had metastatic disease at baseline (91%) and the remaining had locally advanced pancreatic adenocarcinoma, it is interesting to note that the overall patient population derived an extended survival benefit of approximately 3.4 months when compared to the historical OS data [3,4]. In addition, the 1-year and 2-year OS rates were higher compared to MPACT and ACCORD 11 studies. However, it is premature to speculate if the locally advanced pancreatic adenocarcinoma population has contributed to the OS benefit. The above comparison may be considered as hypothesis generating, but nonetheless, the patient characteristics in the pelareorep single arm phase II study are very similar in terms of demographics and extent of disease, to the much larger patient series (33 patients vs. 171 and 430 patients) from the two randomized registration studies. In addition, gemcitabine was administered at a higher dose in both of the phase III studies than in the pelareorep study (1000 vs. 800 mg/m^2^). The median duration of treatment in the single agent gemcitabine arm was six cycles in the ACCORD11 study, three cycles in the MPACT study, and four cycles in the current pelareorep study.

In a randomized phase II study in patients with metastatic pancreatic adenocarcinoma, paclitaxel, and carboplatin were administered alone (37 patients) or in combination with pelareorep (36 patients) [22]. The median OS in the test arm was 7.3 months (95% CI from 4.8 to 11.2 months) versus the control arm at 8.8 months (95% CI from 6.6 to 11.8 months, *p* = 0.68). The median PFS was 4.9 months (95% CI from 3.0 to 6.3 months) in the test arm versus 5.2 months (95% CI from 2.3 to 6.2 months) in the control arm (*p* = 0.6). Although Noonan et al. [22] found no differences in response rate, PFS, or OS between the two arms, the mature data showed a possible delayed effect on OS, with a divergence of survival curves occurring around year 1, and the strongest efficacy signal for improvement in OS occurring around year 2 in the pelareorep-containing arm in comparison to the control arm (20% vs. 9%, respectively). 

In addition to the Noonan et al. study [22], other pelareorep clinical studies have demonstrated delayed effects in OS, which may result from the immuno-oncolytic activity of pelareorep against the tumor cells. A phase II single arm study enrolled 37 patients with metastatic KRAS- or epidermal growth factor receptor (EGFR)-mutated, treatment-naïve, non-small cell lung cancer [31]. Pelareorep was administered IV with paclitaxel and carboplatin. Thirty-one of the 35 evaluable patients had clinical benefit; the objective response rate was 31% (90% 1-sided lower CI) in comparison with the assumed historical response rate for paclitaxel and carboplatin alone of 20%. The median PFS and OS were 4 months and 13.1 months, respectively, and seven patients (20%) were still alive after a median follow-up of 34.2 months (range 26.9–71.5 months). This median OS suggested a survival benefit from pelareorep when compared to previous studies of chemotherapy-naïve non-small cell lung cancer patients [32]. 

The Canadian Cancer Trials Group (CCTG) presented positive OS data from an open-label, randomized, phase II study assessing the therapeutic combination of IV-administered pelareorep given in combination with paclitaxel versus paclitaxel alone, in patients with advanced or metastatic breast cancer [33]. The 74 patient study, powered to 90% and designed by the CCTG, reported a statistically significant improvement in median OS from 10.4 months on the control arm to 17.4 months on the test arm (hazard ratio 0.65, 80% CI from 0.46 to 0.91, *p* = 0.1), although no corresponding difference in median PFS was seen between the test arm and control arm (3.8 month versus 3.4 months, hazard ratio 1.04, 80% CI from 0.76 to 1.43, *p* = 0.87).

Pharmacodynamic analysis showed reovirus replication within the pancreatic tumor and associated apoptosis in one patient with long-term SD. Although no definitive conclusions can be drawn, this current study is among the first in-human studies to demonstrate that IV-administered pelareorep was present in the post-treatment KRAS-activated pancreatic cancer, indicating the ability of reovirus to penetrate the peritumoral desmoplastic stroma, which is a hallmark of pancreatic cancer and a known barricade against chemotherapy. In addition, the accumulation of reoviral protein was associated with ER stress induction and caspase-3 processing, suggesting that pelareorep and gemcitabine treatment exhibited direct proapoptotic activity against the tumor [34]. 

Upon analysis of a single post-treatment biopsy, a high level upregulation of the immune checkpoint marker PD-L1 was shown using IHC. PDAC tumor cells have been shown to express PD-L1 at a higher level than their nonmalignant counterparts [35]. However, one study with an anti-PD-L1 agent alone did not demonstrate an objective response in pancreatic cancers, which are traditionally nonimmunogenic [36]. This suggests an underlying resistance to anti-PD-L1 agents alone, which productive reoviral infection is potentially able to overcome. The combination of pelareorep with an anti-PD-1 antibody showed augmentation of tumor-specific NK responses and attenuation of tumor-specific immunosuppression, resulting in significant survival benefits in C57BL/6 mice with established SC B16 tumors (melanoma) [23]. Prostate cancers are not responsive to immune checkpoint blockade, as they lack a type I interferon signature, chemokine expression, and have decreased T cell infiltration. A recent study by Annels et al. [37] showed that mice implanted with subcutaneous TRAMP-C2 prostate tumors had prolonged survival when treated with a combination of intratumoral pelareorep and anti-PD-1 compared to either therapy alone. This combination also protected mice from subsequent tumor rechallenge. This data suggests that pelareorep can overcome mechanisms of immunotherapy resistance in prostate cancer. Therefore, this ability of overcoming resistance should be further evaluated in pancreatic cancer by combining anti-PD-L1 agents with pelareorep. 

## 4. Materials and Methods

Eligible adults (age ≥ 18 years of age) had an Eastern Cooperative Oncology Group (ECOG) performance status of 0 to 2, and did not receive previous chemotherapy for metastatic disease, had histologic confirmation of advanced or metastatic adenocarcinoma of the pancreas, and a life expectancy of at least 3 months. Patients had measurable disease per RECIST guidelines (version 1.1) [38]. Patients who had received radiotherapy with or without radiotherapy enhancers (such as low dose 5-fluorouracil) should have had evidence of measurable disease that was not in a previously irradiated field and no continuing acute toxic effects (except alopecia) of any prior radiotherapy. No surgical procedures were allowed at least 28 days prior to study enrollment. 

Eligible patients had a baseline absolute neutrophil count (ANC) ≥ 1.5 × 10^9^ [SI units 10^9^/L], platelets ≥ 100 × 10^9^ [SI units 10^9^/L] (without platelet transfusion), serum creatinine ≤ 1.5 × upper limit of normal (ULN), bilirubin ≤ 1.5 × ULN, AST/ALT ≤ 2.5 × ULN (≤ 5 × ULN if patients have liver metastasis), and negative pregnancy test for females of childbearing potential. 

Patients were excluded from the study if they were receiving concurrent therapy with any other investigational anticancer agent, had a history of, or current evidence of, brain metastasis(es), were on immunosuppressive therapy, or had known human immunodeficiency virus (HIV) infection, or active hepatitis B or C, or were pregnant, or breast-feeding woman. Female patients of childbearing potential must have agreed to use effective contraception or be surgically sterile or postmenopausal. Male patients must have agreed to use effective contraception or be surgically sterile. Patients were excluded if they had clinically significant cardiac disease (New York Heart Association, Class III or IV) including pre-existing arrhythmia, uncontrolled angina pectoris, myocardial infarction 1 year prior to study entry, or grade 2 or higher compromised left ventricular ejection fraction, had dementia, or altered mental status that would prohibit informed consent.

### 4.1. Ethical Considerations

All patients gave their written informed consent before their enrollment to the study. The protocol was approved by the ethics and the scientific committees of the participating institutions on 14 January 2009 (NCT00998322). This study was conducted according to the Helsinki declaration and the guidelines on good clinical practice.

### 4.2. Study Design and Treatment

This was a single arm, open-label, nonrandomized, phase II study. Patients received 800 mg/m^2^ gemcitabine IV over 30 min on Days 1 and 8 and pelareorep at 1 × 10^10^ tissue culture infective dose (TCID)_50_ IV over 60 min on Days 1, 2, 8, and 9, in a three week cycle. On Days 1 and 8, pelareorep was administered immediately after completion of the gemcitabine infusion. The gemcitabine and pelareorep dose levels were chosen based on the preliminary results of a phase I study [27]. The first six patients completed at least three weeks of observation after dosing in cycle 1 before subsequent patients were enrolled. Treatment continued until disease progression or until the occurrence of unacceptable drug-related toxicity not responding to either supportive care or dose reduction. 

### 4.3. Study Endpoints

The primary endpoint was antitumor activity, as measured by clinical benefit rate (CBR), defined as the sum of complete response (CR), partial response (PR), and stable disease (SD) for 12 weeks or more. Secondary endpoints were PFS, OS, safety, and pharmacodynamics evaluation. 

### 4.4. Study Assessments

Prior to study treatment, all patients received baseline history, physical exam, ECOG PS assessment, complete blood cell count (CBC), serum chemistry, liver function tests (LFTs), urinalysis, imaging with CT or magnetic resonance imaging (MRI), HIV, Hepatitis B and C, and relevant tumor markers, such as CA 19-9. Optional tumor biopsies were done prestudy within 14 days of the study treatment and at the most clinically appropriate time after having received at least 2 cycles of therapy in those patients who gave consent for the procedure. The timing of the second biopsy varied to optimize the biological data generated by this assessment. 

On Days 1 and 8 of each cycle, physical exam, vital signs, weight, ECOG, concomitant medication assessment, toxicity assessment, CBC, serum chemistry, LFTs, and relevant tumor markers were performed. Tumor evaluation by CT (or appropriate imaging) was performed every 6 weeks, with assessment as per RECIST guidelines (version 1.1) [38]. Patients were withdrawn from the study for the following reasons: disease progression, adverse event, withdrawal of consent, protocol violation, and/or loss to follow-up, death, or clinician’s decision. After the patient discontinued the study, the patient was followed for one month after the last treatment and subsequently every three months to determine date of progression and survival. If patients had an ongoing adverse event considered as suspected, probably related or definitely related to the investigational treatment, the patient was followed until the adverse event was stable, resolved or deemed to be chronic.

### 4.5. Safety Evaluations

The study utilized CTCAE version 3.0 for adverse event (AE) reporting.

### 4.6. Pharmacodynamic Analysis

If archival tumor tissue was available, all efforts were made to study the molecular signatures of the tumor sample, including KRAS mutation status, through Foundation Medicine genomic profile testing. Patients who consented had optional tumor biopsy performed on therapy to assess reoviral replication. Viral replication was detected using a polyclonal antibody derived from mature reovirus viral capsid proteins [39], as the presence of viral RNA may not necessarily imply infectious virus particles. Further apoptosis and cell death with viral replication was assessed using active caspase-3. Finally, to assess the induction of programmed death ligand 1 (PD-L1), IHC was also performed on tumor specimens. 

### 4.7. Statistical Analysis

Demographic data was displayed and descriptive statistics were used to depict the study population. Efficacy data was tabulated. The phase II study used a Simon-two stage design [40]. The following planning parameters were used: α = 0.05, power = 0.80, *p*_o_ = 0.125, *p*_1_ = 0.30. In the first stage, 17 patients were recruited. Responses were tabulated, indicating tumor type, by the best response (CR, PR, CR + PR, CR + PR + SD) to guide decisions regarding future randomized studies. If <3 responses (defined as CR or PR or SD for 12 weeks or more) were observed, the study would conclude in favor of the null hypothesis (that the combination was inactive) and terminate further accrual. However, if 3 or more responses were observed among the 17 patients, the study would enroll an additional 16 patients for a total of 33 evaluable patients. If the total number of responses as defined above were ≥8 (out of 33), the study would conclude in favor of the alternative hypothesis (the drug combination is active). Otherwise, the study would conclude in favor of the null hypothesis. Descriptive statistics were used to analyze the duration of response and safety. All patients who received at least one dose of pelareorep were included in the safety analysis. 

## 5. Conclusions

The phase II study of pelareorep in combination with gemcitabine in advanced pancreatic adenocarcinoma resulted in a 10.2 months median OS, and 1-and 2-year survival rates of 45% and 24%, respectively. These survival rates are better than those expected with gemcitabine monotherapy and comparable to the results obtained with FOLFIRINOX in this setting. However, given the decreased number and severity of treatment-related adverse events, pelareorep is a better tolerated treatment regimen than the latter. Pelareorep was shown to supplement gemcitabine monotherapy and these results should be validated with a larger randomized clinical trial to remove potential selection bias, which is inherent in a smaller single-arm study as the one we present here. There are currently a growing list of clinical studies addressing the combination of immunotherapy with chemotherapy, poly-ADP ribose polymerase (PARP) inhibitors, oncolytic therapy, or other novel therapies in patients with advanced pancreatic cancer [41]. These findings also have implications in future studies of pelareorep in combination with checkpoint blockade in pancreatic adenocarcinoma, as immuno-oncolytic virus therapy upregulates PD-L1 in the tumor. 

## Figures and Tables

**Table 1:** Patient demographics.

Patient Characteristics	Number or Percentage
Total number	34
Median Age (range), years	66 (48–85)
≥65 years of age, %	53
Sex, %	
Male	53
Female	47
ECOG Performance Status, %	
0–1	94
2	6
Ethnicity, %	
Caucasian	71
Black	12
Hispanic	3
Asian	3
Metastatic disease at baseline, %	91
Site of metastases, %	
Liver	65
Lung	6
Peritoneum	18
Number of metastatic sites, %	
1	35
2	18
3	3
>3	35
Median number of cycles (schedule every 3 weeks)	4
Previous chemotherapy/radiotherapy, %	5
Therapy after disease progression on pelareorep, %	53

**Table 2:** Most commonly identified toxicities for pelareorep in combination with gemcitabine. (>10% of patients).

Toxicity *	Total %	Grade 3%	Grade 4%
**Hematologic**			
Anemia	35	24	3
Neutropenia	32	15	12
Thrombocytopenia	15	6	0
**Non-Hematologic**			
Diarrhoea	24	0	0
Nausea	29	0	0
Vomiting	24	0	0
Fatigue	71	9	0
Chills/Flu-like symptoms	51	0	0
Edema	33	0	0
Fever	56	0	0
AST increased	12	6	0
Anorexia/Weight loss	33	0	0
Dyspnea	50	6	0

* Toxicities are reported as per the National Cancer Institute (NCI) common toxicity criteria 3.0; numbers are % of patients. AST: aspartate aminotransferase.

## Supplementary Materials

The following are available online at http://www.mdpi.com/2072-6694/10/6/160/s1, Figure S1: Effect of liver metastases on the overall survival of patients with advanced pancreatic cancer, Table S1: Genetic aberrations identified in patients with advanced pancreatic cancer.
