# Supplementary material for: A Phase II Study of Pelareorep (REOLYSIN®) in Combination with Gemcitabine for Patients with Advanced Pancreatic Adenocarcinoma
Source: Cancers (Basel). 2018 May 25;10(6):160. doi: 10.3390/cancers10060160 (PMC6025223; doi:10.3390/cancers10060160)
Supplement: Supplementary file 1 [file cancers-10-00160-s001.pdf]

Supplementary Materials: A Phase II Study of Pelareorep (REOLYSIN®) in Combination with Gemcitabine for Patients with Advanced Pancreatic Adenocarcinoma

Table S1. Next generation sequencing (NGS) data on available tissue, site of lesion and overall survival.

Patient #	Genomic Alterations Identified	Specimen Site	OS (months)
0002	KRAS G12D, CCNE1 amplification, CDKN2A p16INK4a R58 and p14ARF P72L, MYC amplification, TP53 R213, ARID 1A loss, SPTA1 R1694H	Pancreas	4.6
0003	MET amplification, TSC2 A678fs 14, TP53 M237I, ARID1A Q185, CTCF R49C	H&N node	1.2
0005	PDGFRB V8231I	Peritoneum	43.3
0014	KRAS G12V, TP53 Y220C	Soft tissue	19.7
0015	KRAS G12D, ARID1A D1850fs4, CDKN2A/B loss, TP53 R213L, GRIN2A R1022C, PAX5 V26G, SMAD4 loss	Pancreas	18.0
0018	EGFR R831H	Pancreas	17.4
0020	KRAS G12D, TP53 G266R	Pancreas	3.8
0021	AKT2 amplification, KRAS G12D, CDKN2A p16INK4a G67fs79 and p14ARF, R28fs52+, TP53 R273C, ACVR1B E240	Pancreas	2.5
0022	KRAS G12V, NF1 R2517-subclonal, PDGFRA E289K, CDKN2A/B loss	Pancreas	28.2
0023	KRAS G12V, CDKN2A p16INK4a H83Y and p14ARF A97V, p16INK4a R80 and p14ARF P94L, TP53 Y126C, GLI1 P53S, RNF43 Q414	Liver	39.8
0025	KRAS G12C, CDKN2A p16INK4a A30_G35del, TP53 R282W, ARID1A G2087R	Pancreas	1.8
0026	KRAS G12D, CDKN2A/B loss, RUNX1 P184fs*28	Pancreas	29.1
0027	KRAS G12D, CDKN2A/B loss, TP53 R248Q, ARID1A deletion exons 12-14, SMAD4 D124fs2	Liver	3.8
0028	KRAS amplification-equivocal G12D, CDKN2A p16INK4a R99fs10 and p14ARF R115fs9+, TP53 P153fs28	Pancreas	4.8
0030	KRAS G12V, CDKN2A/B loss, TP53 splice site 672+2T>C, PRDMM1 E743K	Pancreas	17.5

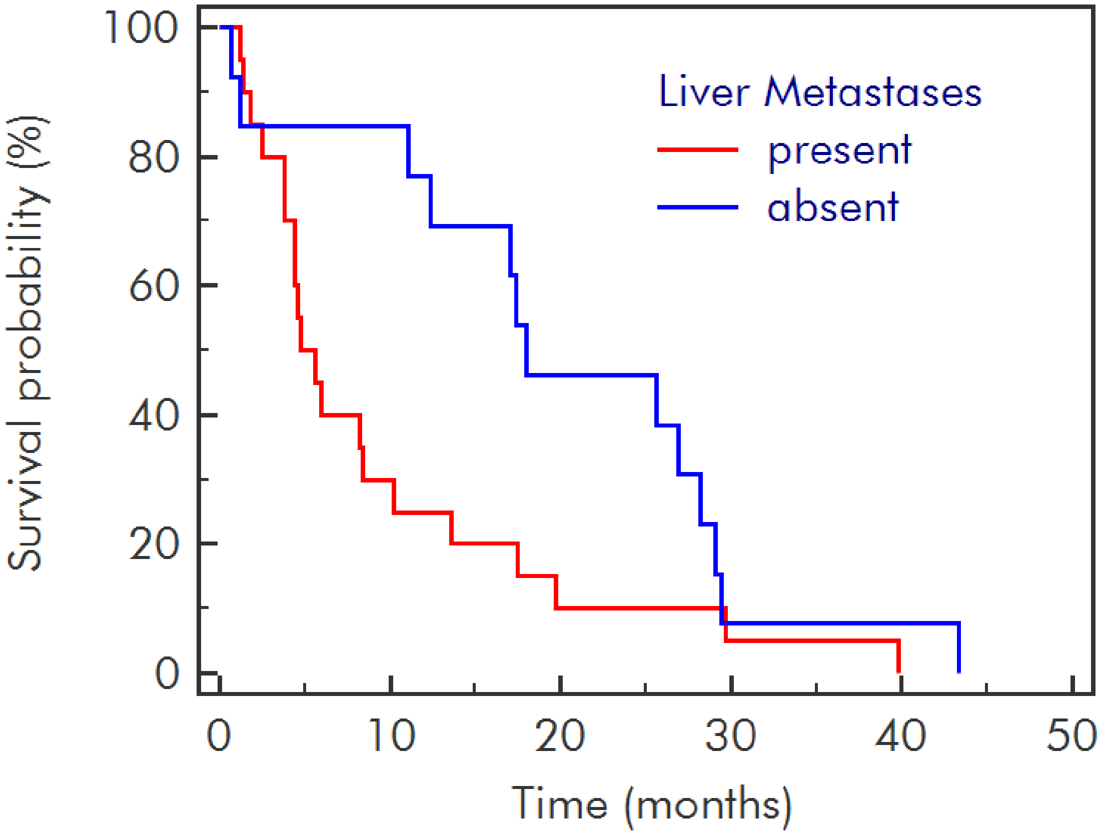

Figure S1. Overall survival of patients with advanced pancreatic cancer based on liver metastasis (present vs absent).
